# Black Tea Extract and Its Theaflavin Derivatives Inhibit the Growth of Periodontopathogens and Modulate Interleukin-8 and β-Defensin Secretion in Oral Epithelial Cells

**DOI:** 10.1371/journal.pone.0143158

**Published:** 2015-11-18

**Authors:** Telma Blanca Lombardo Bedran, Marie-Pierre Morin, Denise Palomari Spolidorio, Daniel Grenier

**Affiliations:** 1 São Paulo Dental School, UNINOVE University, São Paulo, Brazil; 2 Oral Ecology Research Group, Faculty of Dentistry, Laval University, Quebec City, QC, Canada; 3 Department of Oral Diagnosis and Surgery, Araraquara Dental School, State University of São Paulo, São Paulo, Brazil; University of Kiel, GERMANY

## Abstract

Over the years, several studies have brought evidence suggesting that tea polyphenols, mostly from green tea, may have oral health benefits. Since few data are available concerning the beneficial properties of black tea and its theaflavin derivatives against periodontal disease, the objective of this study was to investigate their antibacterial activity as well as their ability to modulate interleukin-8 and human β-defensin (hBD) secretion in oral epithelial cells. Among the periodontopathogenic bacteria tested, *Porphyromonas gingivalis* was found to be highly susceptible to the black tea extract and theaflavins. Moreover, our data indicated that the black tea extract, theaflavin and theaflavin-3,3’-digallate can potentiate the antibacterial effect of metronidazole and tetracycline against *P*. *gingivalis*. Using lipopolysaccharide-stimulated oral epithelial cells, the black tea extract (100 μg/ml), as well as theaflavin and theaflavin-3,3’-digallate (50 μg/ml) reduced interleukin-8 (IL-8) secretion by 85%, 79%, and 86%, respectively, thus suggesting an anti-inflammatory property. The ability of the black tea extract and its theaflavin derivatives to induce the secretion of the antimicrobial peptides hBD-1, hBD-2 and hBD-4 by oral epithelial cells was then evaluated. Our results showed that the black tea extract as well as theaflavin-3,3’-digallate were able to increase the secretion of the three hBDs. In conclusion, the ability of a black tea extract and theaflavins to exert antibacterial activity against major periodontopathogens, to attenuate the secretion of IL-8, and to induce hBD secretion in oral epithelial cells suggest that these components may have a beneficial effect against periodontal disease.

## Introduction

Periodontal disease is a chronic inflammatory disorder of bacterial origin, whose progression and severity is largely modulated by the host immune response [1]. Severe forms of the disease, such as chronic and aggressive periodontitis, lead to destruction of the tooth-supporting tissues, including the periodontal ligament and the alveolar bone and may result in tooth loss [1]. Moreover, periodontitis can influence systemic health by increasing the risk for cardiovascular disease, rheumatoid arthritis, pre-term low birth weight babies, diabetes, and aspiration pneumonia [2]. Periodontitis, which often evolves from untreated gingivitis, a reversible inflammatory condition of the gingiva, is caused by a limited number of Gram-negative anaerobic bacteria that increase in numbers in diseased periodontal sites to the detriment of beneficial bacteria, mostly Gram-positive [3]. While chronic periodontitis has been associated with the presence of the red complex bacteria (*Porphyromonas gingivalis*, *Tannerella forsythia* and *Treponema denticola*), *Aggregatibacter actinomycetemcomitans* is considered as the key etiologic agent of aggressive periodontitis [4, 5]. Periodontopathogens found established in the subgingival biofilm induce host cellular and humoral responses [6]. In most cases, these responses result in the elimination or the control of the pathogens and prevent the establishment and progression of periodontal diseases [6]. However, in other cases, the continuous challenges to the host immune system by periodontopathogens and their virulence factors initiate a number of host-mediated destructive processes that modulate the progression and severity of the disease [7, 8]. Epithelial cells are a major physical barrier to periodontopathogens [9]. They form an integral part of the innate immune system through the secretion of human β-defensins [10] and participate in the inflammatory response of gingival tissues [11]. Previous studies have shown that periodontopathogens can induce the production of proinflammatory cytokines such as interleukin-8 by oral epithelial cells [12].

The conventional treatment of periodontitis relies on the mechanical removal of the bacterial biofilm and toxins from the periodontal pockets by scaling and root planning [1]. In case of poor response to this mechanical periodontal therapy, an adjunctive treatment using systemic or locally applied antimicrobial agents may be appropriate [13]. In this regard, plant polyphenols have been proposed as promising new molecules for adjunctive periodontal therapy [14, 15]. Teas, derived from the leaves of *Camellia sinensis*, can be classified into four distinctive types (white, green, oolong, and black) that differ in terms of manufacturing procedure and chemical composition [16]. Green tea has a high content of catechins, including epigallocatechin-3-gallate (EGCG), whereas black tea which undergoes oxidation, polymerization and other modifications of original components during processing contains theaflavins (benzotropolone dimers of catechins) and their gallate esters [17]. While green tea is largely consumed in East and South East Asia, black tea accounts for approximately 78% of world tea production and is mostly consumed in North America, Europe, and North Africa [18]. The literature has reported many beneficial effects associated to tea polyphenols in regard to the prevention of various diseases including cancer, coronary heart disease, atherosclerosis, stroke, and intestinal inflammation [19–21]. These health benefit effects have been mostly attributed to the antioxidant activity of tea polyphenols that exert their protective effect by neutralizing the free radicals [18].

Several studies have brought evidence suggesting that tea polyphenols may have oral health benefits [18–21]. However, these studies mostly focused on green tea [22–25]. More specifically, epidemiological and clinical investigations suggested that green tea consumption may have potential oral health benefits thus resulting in a decreased incidence/severity of dental caries and periodontal diseases, the two most common oral bacterial infections. Given that few data are available concerning the beneficial properties of black tea and its theaflavin derivatives against periodontal disease, the objective of this study was to investigate their antibacterial activity as well as their ability to modulate interleukin-8 and human β-defensin (hBD) secretion in oral epithelial cells.

## Materials and Methods

### Black tea extract and theaflavins

The commercial black tea extract (Organic Herb Inc., Changsha, China) used in this study had a theaflavin content of 40.23%, according to the company’s data sheet. A stock solution of the black tea extract was freshly prepared by dissolving 40 mg of powder in one ml of sterile warm distilled water prior to sterilize by filtration (0.2-μm pore size polyethersulfone membrane). Theaflavin and theaflavin-3,3`-digallate, two important components of the black tea extract, were purchased from Chromadex Inc. (Irvine, CA, USA) and prepared in 95% ethanol at a concentration of 10 mg/ml. In some analysis, EGCG **(**Sigma-Aldrich Canada Ltd., Oakville, ON, Canada) was included as control. This major green tea catechin was prepared in 95% ethanol at a concentration of 10 mg/ml.

### Determination of minimal inhibitory concentration and minimal bactericidal concentration values

*P*. *gingivalis* ATCC 33277, *Fusobacterium nucleatum* ATCC 25586, *Prevotella intermedia* 5W2, and *A*. *actinomycetemcomitans* ATCC 29522 were grown in Todd Hewitt broth (Becton, Dickinson and Company, Sparks, MD, USA) supplemented with 0.001% hemin and 0.0001% vitamin K (THB-HK). Cultures were incubated at 37°C in an anaerobic chamber (N_2_:H_2_:CO_2_ / 75:10:15). The minimal inhibitory concentration (MIC) values of black tea extract, theaflavin, and theaflavin-3,3`-digallate for the above bacteria were determined by a broth microdilution assay. Briefly, 24-h cultures were diluted in fresh THB-HK to obtain an optical density at 660 nm (OD_660_) of 0.2. Equal volumes (100 μl) of bacteria and serial two-fold dilutions of compounds under investigation in culture medium were mixed into the wells of a 96-well microplate. Control wells with no bacteria were also prepared. A control using only the carrier solvent (ethanol) was also performed and showed that at the dilution used, it has no effect on bacterial growth (data not shown). Tetracycline was used as a reference antibiotic. After an incubation of 24 h at 37°C under anaerobic conditions, bacterial growth was recorded visually. MIC values (μg/ml) corresponded to the lowest concentrations of compounds at which no bacterial growth occurred. To determine minimal bactericidal concentration (MBC) values, aliquots (10 μl) from each well with no visible growth were spread on solid culture plates, which were incubated for five days at 37°C under anaerobic conditions. MBC values (μg/ml) were defined as the lowest concentrations at which no colonies grew. The MIC and MBC assays were performed in triplicate and were repeated three times. Representative data are presented.

### Synergistic effect of the black tea extract and theaflavins in combination with metronidazole or tetracycline

The ability of the black tea extract, theaflavin, and theaflavin-3,3`-digallate to potentiate the antibacterial effect of metronidazole or tetracycline against *P*. *gingivalis* ATCC 33277 was assessed using the checkerboard technique [26]. The above compounds were serially diluted in THB-HK (100 μl) along the ordinate of a 96-well microplate, while the antibiotics were serially diluted in THB-HK (100 μl) along the abscissa. Suspensions of *P*. *gingivalis* prepared in THB-HK and adjusted to an OD_660_ of 0.2 were used as the inoculum. The wells were inoculated with 100 μl of the bacterial suspensions, and the microplate was incubated for 24 h at 37°C under anaerobic conditions. Wells with no bacteria or compounds were used as controls. Bacterial growth was assessed visually. The lowest concentration at which no visible growth occurred was considered the MIC value. The fractional inhibitory concentration index (FICI) was calculated using the following equation: FICI = FIC_A_ + FIC_B_ = (MIC_Black tea extract or theaflavin or theaflavin-3,3’-digallate_ in combination/MIC_Black tea extract or theaflavin or theaflavin-3,3’-digallate_ alone) + (MIC_Antibiotic_ in combination/MIC_Antibiotic_ alone). An FICI < 0.5 was considered as indicating a synergistic effect, an FICI ≥ 0.5 and < 1.0 as indicating a partial synergy, an FICI ≥ 1 and < 2.0 as indicating an additive effect, an FICI ≥ 2.0 and < 4.0 as indicating no effect, and an FICI ≥ 4.0 as indicating an antagonistic effect. Assays were performed in triplicate for a minimum of three independent experiments to ensure reproducibility. A representative set of data is presented.

### Cultivation of oral epithelial cells and determination of cytotoxicity

The immortalized human oral epithelial cell line OBA-9, which was kindly provided by M. Mayer (Department of Microbiology, Institute of Biomedical Sciences, University of São Paulo, São Paulo, Brazil), was cultured in keratinocyte serum-free medium (K-SFM; Life Technologies Inc., Burlington, ON, Canada) containing the growth factor supplement provided (insulin, epidermal growth factor, fibroblast growth factor) and 100 μg/ml of penicillin G-streptomycin at 37°C in a 5% CO_2_ atmosphere until they reached confluence. The effects of the black tea extract, theaflavin, and theaflavin-3,3`-digallate on oral epithelial cell viability was determined with a colorimetric MTT cell viability assay (Roche Diagnostics, Mannheim, Germany), using 3-[4,5-diethylthiazol- 2-yl]-2,5-diphenyltetrazolium bromide as the substrate.

### Production of IL-8 by lipopolysaccharide-stimulated oral epithelial cells

The oral epithelial cells (OBA-9) were seeded in a 12-well microplate (1 ml/well, 1 x 10^6^ cells/ml), and incubated overnight at 37°C in a 5% CO_2_ atmosphere to allow cell adhesion. Thereafter, epithelial cells were pre-treated for 2 h with the black tea extract (100 and 200 μg/ml), theaflavin, theaflavin-3,3’-digallate or EGCG (10 and 50 μg/ml) prior to being stimulated with *A*. *actinomycetemcomitans* ATCC 29522 lipopolysaccharide (LPS) (1 μg/ml) for 24 h at 37°C in a 5% CO_2_ atmosphere. The supernatants were collected, centrifuged (500 x g for 5 min at 4°C), and stored at -20°C until used for quantification of IL-8 using a commercial enzyme-linked immunosorbent assay (ELISA) kit (eBioscence Inc., San Diego, CA, USA) according to the manufacturer’s protocol. OBA-9 cells incubated in the absence of tea compounds but in the presence of the carrier solvent (ethanol) at the corresponding dilution showed that it has no effect on IL-8 secretion.

### Production of hBD-1, hBD-2 and hBD-4 by oral epithelial cells

OBA-9 cells were treated with the black tea extract (150 and 200 μg/ml), theaflavin (75 and 100 μg/ml), theaflavin-3,3’-digallate (75 and 100 μg/ml) or EGCG (100 μg/ml) for 48 h at 37°C in a 5% CO_2_ atmosphere. Following stimulation, the supernatants were collected, centrifuged (500 x g for 5 min) and stored at -80°C until used. Unstimulated epithelial cells were used as a control. Commercial ELISA kits (PeproTech, Rocky Hill, NJ, USA) were used to quantify hBD-1 hBD-2, and hBD-4 concentrations in the cell-free supernatants, according to the manufacturer’s protocol. OBA-9 cells incubated in the absence of tea compounds but in the presence of the carrier solvent (ethanol) at the corresponding dilution showed that it has no effect on hBD secretion (data not shown).

### Statistical analysis

Unless indicated otherwise, assays were performed in triplicate and the means ± standard deviations were calculated. Experiments were carried out a minimum of three times to ensure reproducibility, and data from a representative experiment are presented. Differences between means were analyzed for statistical significance using a one-way ANOVA.9

## Results

The antibacterial activity of the black tea extract, theaflavin, and theaflavin-3,3’-digallate was determined against four periodontopathogenic bacterial species. As reported in Table 1, the MIC values of the black tea extract were in the range of 500 to 2000 μg/ml, while the MBC values were in the range of 1000 to 8000 μg/ml. Both theaflavin and theaflavin-3,3’-digallate showed antibacterial activity against the four periodontopathogenic bacteria tested with MIC values in the range of 125 to 500 μg/ml. The most susceptible bacteria to the black tea extract and theaflavins were the two black-pigmented bacteria *P*. *gingivalis* and *P*. *intermedia*. The reference antibiotic tetracycline showed lower values of MIC (0.20–1.56 μg/ml) and MBC (3.13–25 μg/ml) against the four periodontopathogenic bacterial species.

**Table 1:** Minimal inhibitory concentration (MIC) and minimal bactericidal concentration (MBC) values of the black tea extract, theaflavin, theaflavin-3,3’-digallate, and tetracycline against four periodontopathogenic bacterial species.

	MIC/MBC values (μg/ml)			
Strain	Black tea extract	Theaflavin	Theaflavin-3,3’-digallate	Tetracycline
*P*. *gingivalis*	500/1000	125/500	250/500	0.78/12.5
*P*. *intermedia*	500/1000	125/1000	125/500	0.20/3.13
*F*. *nucleatum*	2000/4000	250/>1000	250/>1000	0.39/25
*A*. *actinomycetemcomitans*	2000/8000	250/>1000	500/1000	1.56/25

Since combination therapy can result in synergistic interactions between drugs, we further investigated the effect of the black tea extract, theaflavin, and theaflavin-3,3’-digallate on *P*. *gingivalis* ATCC 33277 by testing their ability to potentiate the antibacterial activity of metronidazole and tetracycline, two conventional antibiotics currently used in adjunctive periodontal therapy. As reported in Table 2, a synergistic effect was observed when the black tea extract, theaflavin or theaflavin-3,3’-digallate was used in combination with metronidazole. Regarding tetracycline, synergy was obtained when combined with the black tea extract or theaflavin, while its association with theaflavin-3,3’-digallate resulted in a partial synergistic effect.

**Table 2:** FICI values of the effect of the black tea extract, theaflavin, or theaflavin-3,3’-digallate in combination with metronidazole or tetracycline on *P*. *gingivalis* ATCC 33277.

Combination	FICI	Effect
Black tea extract + metronidazole	0.367	Synergy
Theaflavin + metronidazole	0.344	Synergy
Theaflavin-3,3’-digallate + metronidazole	0.458	Synergy
Black tea extract + tetracycline	0.339	Synergy
Theaflavin + tetracycline	0.218	Synergy
Theaflavin-3,3’-digallate + tetracycline	0.708	Partial synergy

**Fig 1:**
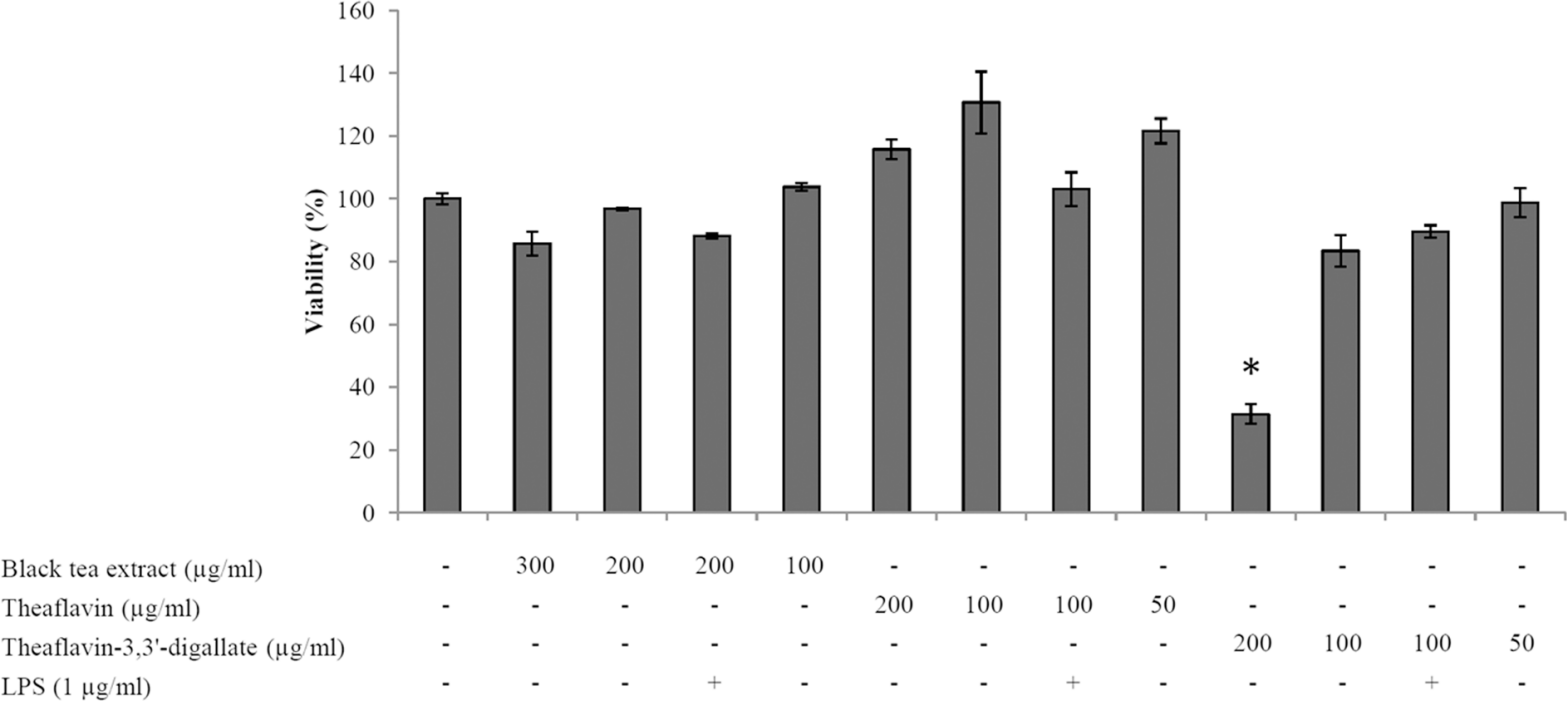
Effects of the black tea extract, theaflavin and theaflavin-3,3’-digallate, individually or in combination with *A*. *actinomycetemcomitans* LPS on the viability of oral epithelial cells. *, significantly different from control at *p* < 0.01.

**Fig 2:**
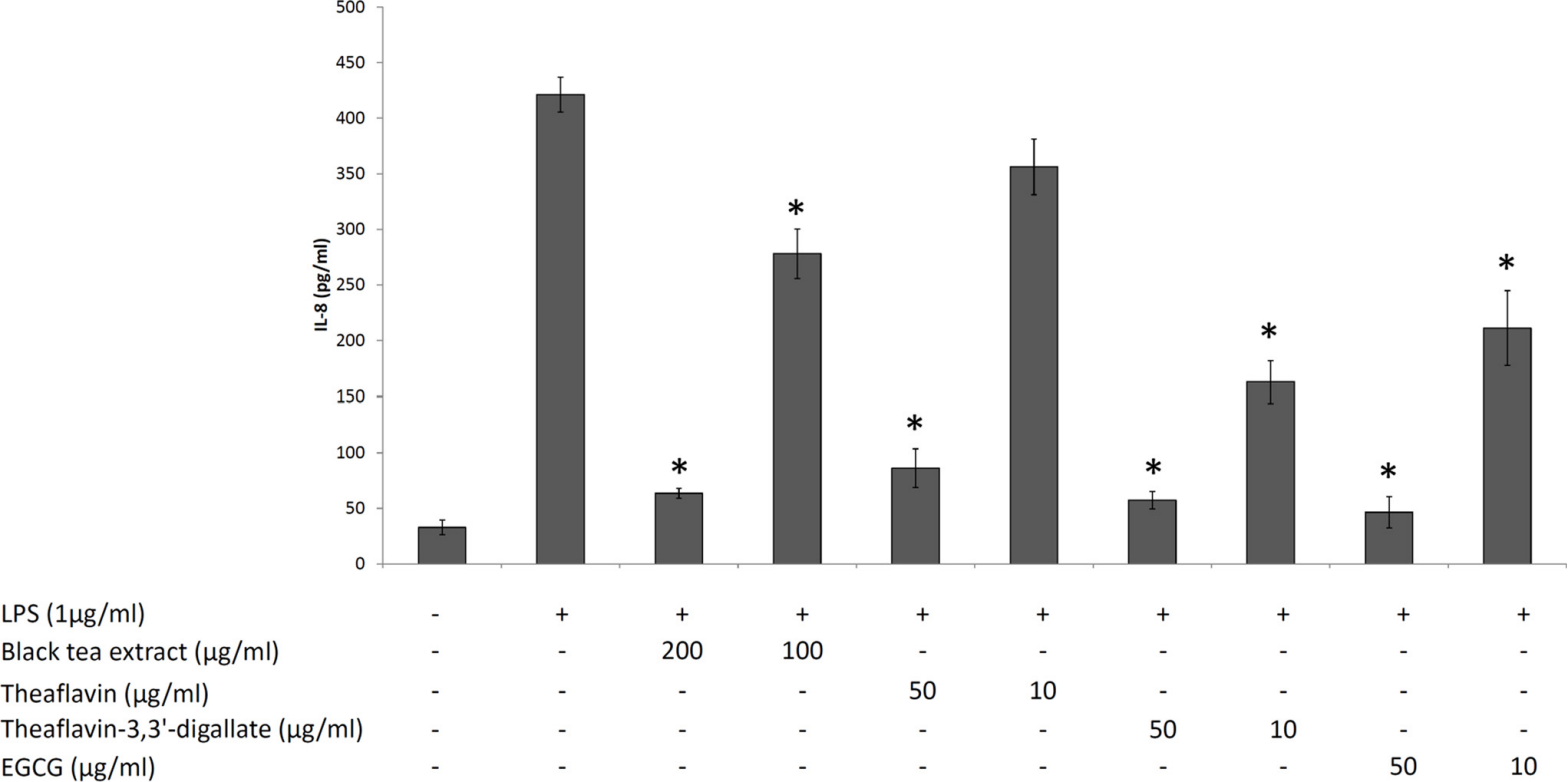
Effects of the black tea extract, theaflavin, theaflavin-3,3’-digallate, and EGCG on the secretion of IL-8 by LPS-stimulated oral epithelial cells. *, significantly different from control (no compounds) at *p* < 0.01.

The effects of the black tea extract, theaflavin, and theaflavin-3,3’-digallate, individually or in association with *A*. *actinomycetemcomitans* LPS, on the viability of oral epithelial cells (OBA-9 cell line) were determined using the colorimetric MTT assay. As shown in Fig 1, with the exception of theaflavin-3,3’-digallate at 200 μg/ml, none of the concentrations of the black tea extract and theaflavins tested, individually or in the presence of LPS, caused a significant decrease of the viability of oral epithelial cells. Non-cytotoxic concentrations were then used to evaluate the ability the black tea extract, theaflavin, and theaflavin-3,3’-digallate to attenuate the secretion of IL-8 by LPS-stimulated oral epithelial cells. As reported in Fig 2, LPS significantly increased the secretion of IL-8 by the oral epithelial cells compared to the unstimulated cell. The secretion of IL-8 was significantly inhibited by all compounds tested, suggesting an anti-inflammatory property. More specifically, the black tea extract (200 μg/ml), as well as theaflavin and theaflavin-3,3’-digallate (50 μg/ml) reduced IL-8 secretion by 85%, 79%, and 86%, respectively. EGCG, the major constituent of green tea used as a control, also significantly attenuated the secretion of IL-8 by the oral epithelial cells.

**Fig 3:**
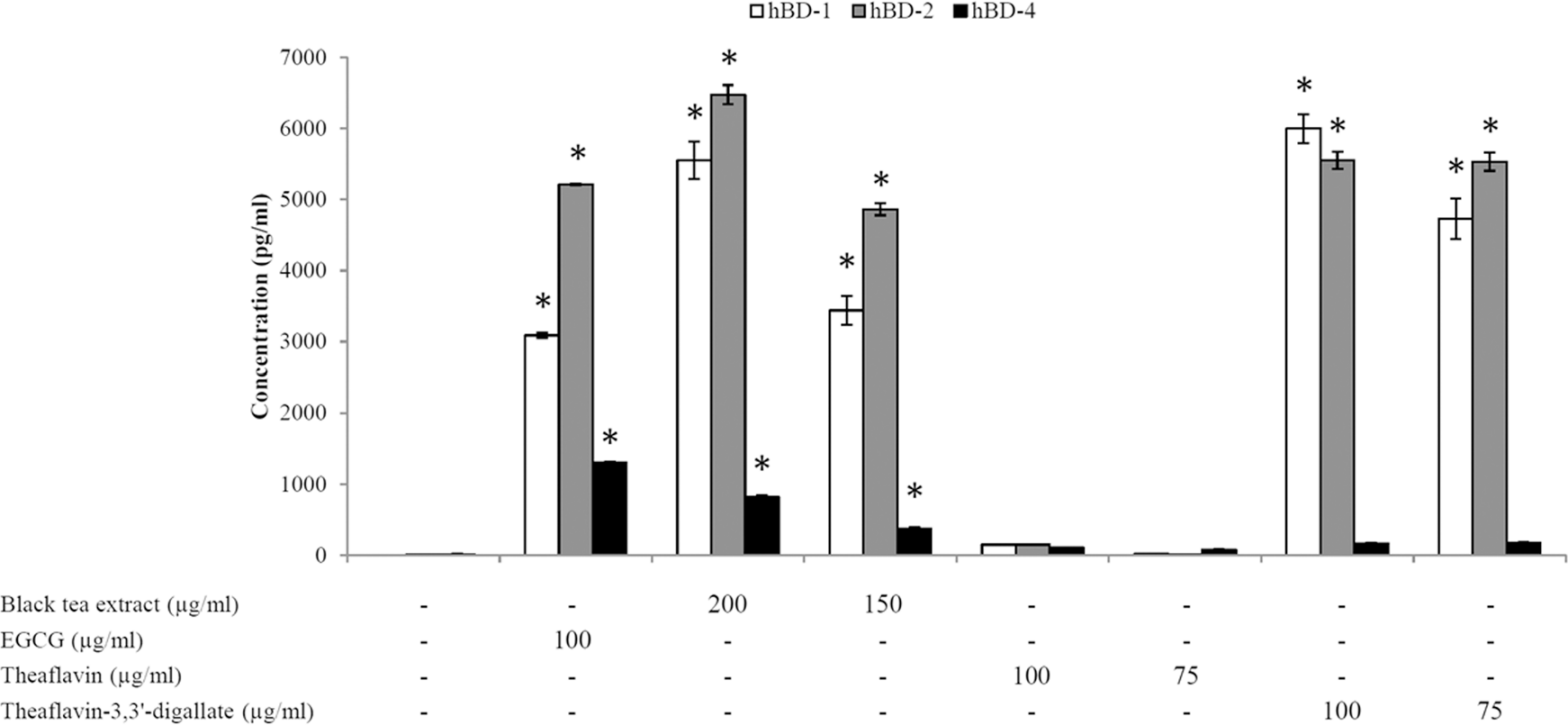
Effects of the black tea extract, theaflavin, theaflavin-3,3’-digallate, and EGCG on the secretion of hBDs by oral epithelial cells. *, significantly different from control (no compounds) at *p* < 0.01.

The ability of black tea polyphenols to induce the secretion of the antimicrobial peptides hBD-1, hBD-2 and hBD-4 by oral epithelial cells (OBA-9) was then evaluated. The secretion of all three hBDs was dose-dependently up-regulated following stimulation (48 h) of the epithelial cells with the black tea extract. At the highest concentration tested (200 μg/ml) of the black tea extract, the epithelial cells secreted 5550 pg/ml of hBD-1, 6477 pg/ml of hBD-2, and 816 pg/ml of hBD-4 (Fig 3). Theaflavin and theaflavin-3.3’-digallate were also tested for their ability to up-regulate hBD secretion. As shown in Fig 3, while theaflavin did not induce the secretion of significant amounts of hBDs by oral epithelial cells, theaflavin-3,3’-digallate at 75 μg/ml and 100 μg/ml was able to significantly induce the secretion of hBD-1 and hBD-2. A treatment of epithelial cells with theaflavin-3,3’-digallate at 100 μg/ml resulted in the secretion of 5997 pg/ml of hBD-1 and 5549 pg/ml of hBD-2. EGCG used as a control significantly increased the secretion of the three hBDs tested.

## Discussion

Epidemiological studies and research findings correlated black tea polyphenols with preventive and therapeutic properties against a variety of human diseases such as cancer, as well as metabolic and cardiovascular diseases [27, 28]. Theaflavins, that is the main polyphenol present in the black tea extract used in this study, are formed by crushing leaves of the plant *C*. *sinensis* and allowing oxidation of the catechins and gallocatechins [27, 28]. While a number of studies evaluated the positive impact of green tea and EGCG for oral health, few reports focused on the beneficial effects of black tea and theaflavins for the prevention and control of oral diseases. It has been suggested that black tea can exert a beneficial effect against dental caries through its high fluoride content [29] and its ability to inhibit salivary amylase activity thus reducing the cariogenic potential of starch [30]. More recently, Kong et al. [31] reported that theaflavins inhibit *P*. *gingivalis* proteinases, in addition to attenuate matrix metalloproteinase (MMP) production by human gingival fibroblasts.

Given that the mechanical periodontal treatment of scaling and root planning is not always sufficient to stop periodontitis progression, the search for new strategies and therapies is of particular interest. In this study, we investigated the capacity of a black tea extract and its theaflavin derivatives to inhibit the growth of major periodontopathogens and to potentiate the antibacterial effect of conventional antibiotics. Moreover, we also evaluated their ability to modulate IL-8 and hBD secretion by oral epithelial cells.

The black tea extract as well as theaflavin and theaflavin-3,3’-digallate were active against *P*. *gingivalis* and *P*. *intermedia* and to a lesser extent against *F*. *nucleatum* and *A*. *actinomycetemcomitans*. While several studies investigated the antibacterial activity of green tea extracts and catechins on oral pathogens, [32–34], few data are available concerning theaflavins. In agreement with our study, Kong et al. [31] used a theaflavin mixture containing theaflavin, theaflavin-3-gallate and theaflavin-3,3’-digallate and found MIC and MBC values of 125 μg/ml and 500 μg/ml, respectively on *P*. *gingivalis*. Tea theaflavins, as for the catechins, are likely to exert their antibacterial effect by interacting with the bacterial membranes and causing irreversible damages. This is supported by a recent study by Sirk et al. [35] who showed that tea theaflavins have an affinity for the bilayer surface via hydrogen bonding.

Combining conventional antibiotics with natural antibacterial agents such as polyphenols may be a valuable strategy to limit the emergence of antibiotic resistance in bacterial pathogens. Our data indicated that the black tea extract, theaflavin and theaflavin-3,3’-digallate can potentiate the antibacterial effect of metronidazole and tetracycline, two antibiotics currently used in adjunctive treatments of periodontitis, against *P*. *gingivalis*. Theaflavins may synergize the effect of these antibiotics by acting on different targets. To the best of our knowledge, only one study previously investigated the synergistic interactions of tea theaflavins with antibiotics [36]. More specifically, Betts et al. [36] reported a significant synergy between theaflavin and ampicillin against *Stenotrophomonas maltophilia*, an opportunistic nosocomial pathogen.

Chemokine production induced by periodontopathogens is believed to play an important role in initiating inflammatory reactions in gingival tissue through migration of polymorphonuclear leukocytes, monocytes, and macrophages to sites of infection [37]. Interestingly, the concentration of IL-8 in the gingival crevicular fluid of inflamed periodontal sites has been correlated with the severity of periodontitis [38]. In addition, periodontal treatment reduces the number of immune cells and the levels of IL-8 infiltrates, suggesting that this chemokine plays a role in periodontal status [39]. The fact that the black tea extract and theaflavins inhibited the secretion of IL-8 by oral epithelial cells stimulated with LPS suggests that they have the potential to reduce the influx of inflammatory cells to diseased sites and the amplification of LPS-induced inflammatory processes. These results are in agreement with the study of Aneja et al. [40] showing that theaflavin significantly attenuated the secretion and gene expression of IL-8 by tumor necrosis factor α-stimulated pulmonary epithelial cells. Evidence were brought that this effect involves the inhibition of activator prorein-1 (AP-1) and IκB kinase (IKK) activation. The anti-inflammatory property of theaflavins has also been demonstrated in human gingival fibroblasts. Hosokawa et al. [41] reported the capacity of theaflavin-3,3’-digallate to reduce the secretion of IL-6 by gingival fibroblasts stimulated with tumor necrosis factor superfamily 14 (TNFSF14). Moreover, a recent study by Kong et al. [31] showed that black tea theaflavins inhibited the secretion and gene expression of MMP-1 and MMP-2 by gingival fibroblasts stimulated with *P*. *gingivalis*.

The gingival epithelium is a stratified squamous tissue that is an interface between the external environment and the underlying periodontal connective tissue [42]. It plays a key role in resisting bacterial infections by acting as a physical barrier against bacteria and mounting an innate immune defense by the secretion of antimicrobial peptides, more specifically β-defensins [10, 43]. These cationic peptides interact with the microbial cell membrane, leading to pore formation and lysis of major periodontopathogens [44]. Our results showed that the black tea extract as well as theaflavin-3,3’-digallate were able to increase the secretion of hBD-1 and hBD-2, and to a lesser extent of hBD-4 by oral epithelial cells. Given that no induction was observed with theaflavin, it appears that the presence of galloyl moieties is critical. Since the above observations were obtained using an immortalized epithelial cell line, further studies should use primary gingival epithelial cells to confirm this property. As previously observed [45], EGCG used as control also induced the secretion of hBDs by oral epithelial cells. The up-regulation of the secretion of hBDs may have several positive impacts on periodontal health. Given that hBDs are active against the major periodontopathogens [44], the ability of the black tea extract and theaflavin-3,3’-digallate to induce hBD secretion by oral epithelial cells may contribute to reduction of bacterial invasion of the connective tissues and prevent periodontal destruction. The fact that anti-inflammatory and wound healing properties have recently been associated to hBDs further supports the significance of our data for periodontal health [43, 46, 47].

Results from the present study will serve as the foundation for human clinical trials aimed at demonstrating that the daily intake of black tea or the use of oral-hygiene products (mouthrinses and chewing gums) or slow periodontal-release devices (to be inserted in diseased periodontal sites) containing black tea bioactive molecules may be an economic and safe procedure to maintain periodontal health in the general population.

## Conclusions

The ability of a black tea extract and theaflavins to exert antibacterial activity against major periodontopathogens as well as to attenuate the secretion of IL-8, and to induce hBD secretion in oral epithelial cells suggest that these components may have a beneficial effect on periodontal disease.
